# THE ROLE OF SOCIAL DETERMINANTS OF HEALTH ON CANCER RISK BEHAVIORS AMONG OLDER NIGERIAN MEN

**DOI:** 10.1093/geroni/igad104.0407

**Published:** 2023-12-21

**Authors:** Darlingtina Esiaka, Candidus Nwakasi, Runcie Chidebe

**Affiliations:** University of Kentucky, Lexington, Kentucky, United States; University of Connecticut, Storrs, Connecticut, United States; Miami University, Oxford, Ohio, United States

## Abstract

Despite the growing cancer incidence and mortality rate in Nigeria, there exists prevalence of imminent and aggressive stage of cancer diagnosis among Nigerian men due to cancer risk behaviors and lack of cancer preventive behaviors such as early screening. This adds to the increasing rates of cancer disparities in Nigeria and Sub-Saharan Africa. Research links social determinants of health (SDOH) to myriad of cancer outcomes including cancer detection and prevention behaviors. To contribute to our understanding of the complexity of cancer risk behaviors, this cross-sectional study aimed to determine the relationship demographic (age, education, employment, income), health (cancer knowledge, cancer beliefs, illness attitudes) and social determinants of health (health insurance, public health infrastructure, rural vs. urban dwelling) have on cancer risk behaviors among older Nigerian men (N=411, age 50+). Results show a significant association between SDOH and cancer risk behaviors. Lack of insurance, absence of health infrastructures, and rural dwelling were correlates of cancer risk behaviors. Also, cancer beliefs, illness attitude, income, and employment had significant relationships with cancer risk behaviors. Our findings point to the detrimental effects of these factors on the fight against cancer disparities. Understanding the mechanisms by which older Nigerian men experience health, requires efforts in identifying constructs that influence their daily-lived experiences and their impact on health-related behaviors. Our data can inform global health actions to eliminate cancer disparities at the individual, organization, and government levels.

